# Investigation of Appropriate Scaling of Networks and Images for Convolutional Neural Network-Based Nerve Detection in Ultrasound-Guided Nerve Blocks

**DOI:** 10.3390/s24113696

**Published:** 2024-06-06

**Authors:** Takaaki Sugino, Shinya Onogi, Rieko Oishi, Chie Hanayama, Satoki Inoue, Shinjiro Ishida, Yuhang Yao, Nobuhiro Ogasawara, Masahiro Murakawa, Yoshikazu Nakajima

**Affiliations:** 1 Department of Biomedical Informatics, Institute of Biomaterials and Bioengineering, Tokyo Medical and Dental University, Tokyo 101-0062, Japan; onogi.bmi@tmd.ac.jp (S.O.); nakajima.bmi@tmd.ac.jp (Y.N.); 2 Department of Anesthesiology, Fukushima Medical University, Fukushima 960-1295, Japan; rieponko@fmu.ac.jp (R.O.); hanayamachie8780@yahoo.co.jp (C.H.); seninoue@fmu.ac.jp (S.I.); murakawa@fmu.ac.jp (M.M.); 3 TCC Media Lab Co., Ltd., Tokyo 192-0152, Japan; s-ishida@kikuchiseisakusho.co.jp (S.I.); nobuhiro.ogasawara@kikuchiseisakusho.co.jp (N.O.); 4 IOT SOFT Co., Ltd., Tokyo 103-0023, Japan; yo.uko@iot-soft.co.jp

**Keywords:** ultrasound image sensing, nerve detection, convolutional neural networks, ultrasound-guided nerve block anesthesia

## Abstract

Ultrasound imaging is an essential tool in anesthesiology, particularly for ultrasound-guided peripheral nerve blocks (US-PNBs). However, challenges such as speckle noise, acoustic shadows, and variability in nerve appearance complicate the accurate localization of nerve tissues. To address this issue, this study introduces a deep convolutional neural network (DCNN), specifically Scaled-YOLOv4, and investigates an appropriate network model and input image scaling for nerve detection on ultrasound images. Utilizing two datasets, a public dataset and an original dataset, we evaluated the effects of model scale and input image size on detection performance. Our findings reveal that smaller input images and larger model scales significantly improve detection accuracy. The optimal configuration of model size and input image size not only achieved high detection accuracy but also demonstrated real-time processing capabilities.

## 1. Introduction

Ultrasound imaging is a crucial modality in medical sensing which is widely utilized for visualizing organs, tissues, and lesions within the body. Its ability to achieve real-time, continuous, and noninvasive imaging is invaluable not only for diagnostic purposes but also for guiding medical procedures such as anesthesia administration, biopsies, and surgeries. In anesthesiology, ultrasound plays a pivotal role in various applications, including regional anesthesia, chronic pain interventions, vascular access, airway assessments, pneumonic and gastric ultrasounds, and neuromonitoring [1]. Particularly, ultrasound-guided peripheral nerve blocks for regional anesthesia have emerged as a prominent application, offering direct visualizations of nerves and their surrounding anatomical structures, thereby enhancing the accuracy and efficacy of anesthesia. Despite its benefits, ultrasound imaging encounters challenges such as speckle noise and acoustic shadows that can degrade image quality and obscure anatomical boundaries. Additionally, nerve regions in ultrasound images are typically small and exhibit considerable variability in shape across different patients. The appearance of ultrasound images can also vary with patient-specific characteristics and probe-manipulation techniques. These complexities make it difficult for even experienced anesthesiologists to accurately identify nerve regions in ultrasound images [2]. Consequently, there is a pressing need for tools that assist anesthesiologists in accurately localizing nerve tissues while performing ultrasound-guided peripheral nerve blocks (US-PNBs).

Numerous studies have explored the automated detection and segmentation of nerve tissues from ultrasound images [3]. To address noise and variability, Hadjerci et al. [4,5] proposed combined approaches involving denoising preprocessing, machine learning, and active contour techniques for nerve segmentation in ultrasound images. In recent years, deep convolutional neural networks (DCNNs) have gained traction for their ability to automatically extract relevant image features and capture intricate patterns, surpassing conventional machine learning techniques in nerve detection and segmentation from ultrasound images [6]. Many studies have employed DCNNs, which delineate nerves through pixel-wise labeling to help doctors not only localize nerves but also diagnose nerve disorders, for segmentation [2,7,8,9,10,11,12,13,14,15,16,17,18,19,20]. Encoder–decoder networks based on U-Net [21] or two-stage networks based on Mask R-CNN [22] are popular and have demonstrated strong performance in ultrasound image segmentation tasks. Tian et al. [16] conducted a comparative study of various DCNN models for brachial plexus nerve trunk segmentation from ultrasound images, revealing that U-Net achieved the best segmentation performance. Ding et al. [2] developed a multi-object assistance-based brachial plexus segmentation network (MallesNet) derived from Mask R-CNN to achieve better segmentation accuracy than U-Net and other variants. On the other hand, some studies have utilized DCNNs for object detection which output the position coordinates and sizes of bounding boxes as well as their classes to localize anatomical structures of interest using rectangles [23,24]. Alkhatib et al. [24] combined a 2D DCNN for object detection with a 1D DCNN functioning as a texture descriptor to improve nerve detection performance in ultrasound images.

This study focuses on assisting anesthesiologists in accurately localizing nerve tissues during US-PNB, a task achievable even with DCNNs designed for object detection. Since DCNNs for segmentation can delineate more detailed shapes of anatomical structures but require a large computational cost to output pixel-wise labeled results, we employ a DCNN for object detection that eliminates the computational cost of pixel-wise labeling and spends it on extracting and refining multi-scale image features. Hence, this study presents automatic and accurate nerve detection from ultrasound images using Scaled-YOLOv4 [25], a more scalable DCNN model that provides a superior trade-off between speed and accuracy for real-time object detection compared to the DCNN models used in previous studies [21,22,23,24]. Furthermore, although it is known that the choice of model size (i.e., the number of convolutional layers and filters) and input image size significantly affect detection accuracy in DCNN-based object detection [25,26], the appropriate model size and image size for ultrasound images remain unclear. In particular, for ultrasound images containing speckle noise and acoustic shadows, the receptive field size and input image size of the DCNN might cause it to be strongly affected by such noise, making it challenging to train and capture critical image features necessary for detecting target anatomical structures. To minimize the negative effects of noise and accurately capture the image features of the target anatomical structures, model scaling and image scaling are crucial elements. Therefore, this study also investigates appropriate model scaling and input image scaling for DCNN-based nerve detection from ultrasound images.

The contributions of this study include applying a one-stage DCNN detector with adjustable accuracy and speed through model scaling and image scaling to nerve detection in ultrasound images, evaluating its performance and feasibility for real-time US-PNB support, and identifying the optimal combination of model scaling and image scaling for nerve detection in ultrasound images.

## 2. Materials and Methods

### 2.1. Dataset

In this study, we utilized two datasets to investigate the efficacy of DCNN-based nerve detection in ultrasound images for supporting US-PNB procedures.

**Figure 1:**
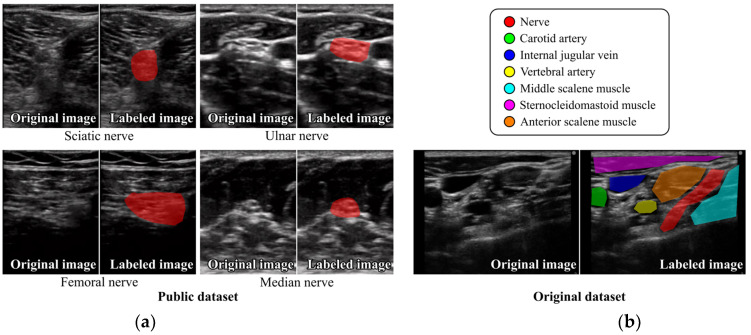
Examples of ultrasound images and their corresponding labeled images in (**a**) Public dataset and (**b**) Original dataset.

The first dataset, referred to as the “Public dataset”, is a publicly available dataset for nerve segmentation in ultrasound images accessible through Kaggle datasets [27]. This dataset consists of 619 ultrasound images, including images of the sciatic nerve (287 cases), ulnar nerve (221 cases), femoral nerve (70 cases), and median nerve (41 cases), along with their corresponding labeled images. Each labeled image contains only one nerve. The images, acquired with a resolution of 640 × 480 pixels using a SONOSITE Nano-Maxx device (FUJIFILM Sonosite, Inc., Bothell, WA, USA) by the Universidad Tecnológica de Pereira and Santa Mónica Hospital, Dosquebradas, Colombia, were annotated by an anesthesiologist from the Santa Mónica Hospital. The ultrasound images and labeled images were subsequently cropped to a region of interest measuring 360 × 279 pixels after improving the annotation using morphological operations of dilation and erosion [12]. Figure 1a shows an example from the Public dataset.

The second dataset, referred to as the “Original dataset”, comprised 993 ultrasound images of the brachial plexus and their corresponding labeled images; these images were obtained from 101 healthy volunteers. The ultrasound images were collected using a SonoSite Edge (FUJIFILM Medical Co., Ltd., Tokyo, Japan) with a resolution of 1024 × 768 pixels by experienced anesthesiologists from Fukushima Medical University during ultrasound procedures for the interscalene approach to brachial plexus block. The labeled images included not only nerves but also surrounding structures such as blood vessels (carotid artery, internal jugular vein, and vertebral artery) and muscles (middle scalene muscle, sternocleidomastoid muscle, and anterior scalene muscle) to enhance the discrimination performance of DCNNs for nerve tissues by providing contextual information about the surrounding tissues. Unlike the Public dataset, this dataset includes ultrasound images that contain multiple anatomical structures or multiple instances of the same anatomical structure. Annotations were manually performed on the ultrasound images by the anesthesiologists using proprietary annotation software, which is not publicly available, developed by IOT SOFT Co., Ltd. to facilitate the annotation process. Similar to the Public dataset, the images in the Original dataset were cropped to regions of interest with sizes of 540 × 753, 600 × 800, and 605 × 710 pixels. Figure 1b shows an example from the Original dataset. Ethical approval for the use of the Original dataset was granted by the Ethical Review Boards of both Fukushima Medical University and Tokyo Medical and Dental University, and written informed consent was obtained from all subjects.

### 2.2. Deep Convolutional Neural Network-Based Nerve Detection

The aim of this study is to assist anesthesiologists in identifying nerves on ultrasound images for safe US-PNB procedures. Given this objective, a detailed pixel-wise segmentation of the target regions is unnecessary. It is sufficient to indicate the approximate locations of the target regions using bounding boxes. Therefore, we focused on DCNNs for object detection based on bounding box regression.

#### 2.2.1. Network Architecture

**Figure 2:**
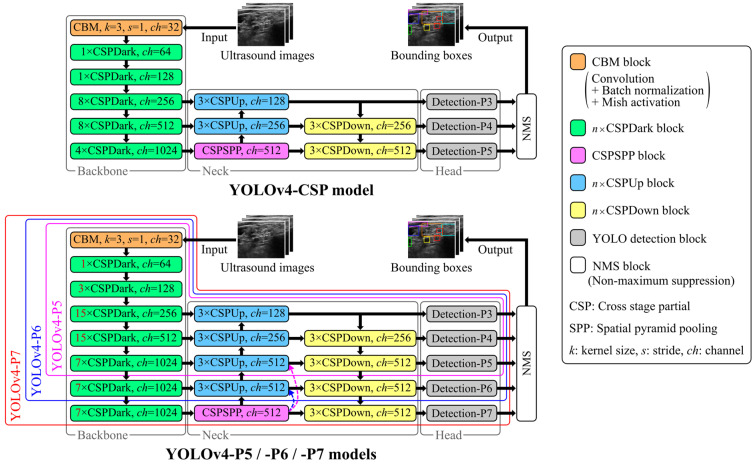
An overview of the Scaled-YOLOv4 models used in this study. The pink and blue dashed arrows indicate replacing the corresponding CSPUp block with a CSPSPP block for YOLOv4-P5 and -P6, respectively.

**Figure 3:**
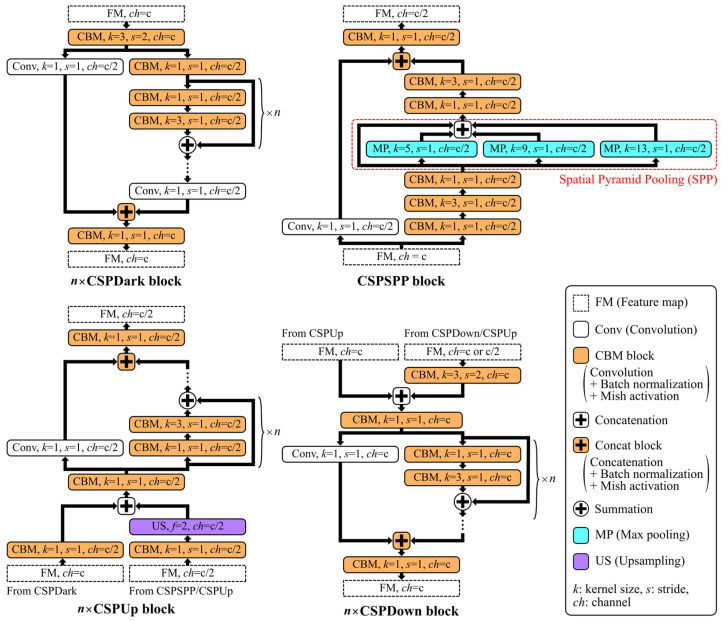
Computation blocks in the Scaled-YOLOv4 models.

In this study, we utilized Scaled-YOLOv4 [25], which was designed to balance speed and accuracy, for the real-time detection of anatomical structures in ultrasound images. Scaled-YOLOv4 is an improved version of YOLOv4 [28], a derivative of the one-stage detector YOLO [29]. Scaled-YOLOv4 models include network architectures with different scaling factors to allow for the selection of the appropriate model based on speed and accuracy requirements. Model scaling, which involves adjusting the number of convolutional layers and the filters in a convolutional layer, is crucial for enhancing DCNN performance. Thus, we used Scaled-YOLOv4 models with different scaling factors, YOLOv4-CSP, -P5, -P6, and -P7, to determine the appropriate model architecture. These models have deeper scaling in the order of YOLOv4-CSP, -P5, -P6, and -P7. Figure 2 presents an overview of the network architectures used in this study. The DCNNs for object detection consist of a backbone for extracting essential image features, a neck for refining the features, and a head for predicting bounding boxes based on the refined features. Final predictions are obtained using non-maximum suppression, which eliminates redundant bounding boxes. The Scaled-YOLOv4 models incorporate cross-stage partial (CSP) [30] architectures in both the backbone and the neck. This configuration reduces computational complexity while preserving accuracy and supports extensive model scaling. Figure 3 depicts the computational blocks in the backbones and necks of the Scaled-YOLOv4 models. CSP architectures, which bifurcate the image feature maps and perform convolutional processing on one of them, are used in all computational blocks.

#### 2.2.2. Training and Prediction

For DCNN-based nerve detection, we generated circumscribed rectangles around labeled anatomical structures from annotated data which served as label data for training. Additionally, since image scaling influences detection performance, as with model scaling [25,26], we resized the input images to 384 × 384, 640 × 640, 896 × 896, and 1152 × 1152 pixels with zero padding to preserve their aspect ratios, investigating the appropriate input image size. For training, DCNNs with different scaling factors were initialized with pre-trained weights on ImageNet [31] and trained using the resized images. The loss function, similar to YOLOv4 [28], included CIoU loss [32] for bounding box regression and cross-entropy loss for classification and confidence. Data augmentation techniques, such as translation, scaling, left–right flip, and mix-up techniques [33], were employed to train DCNNs with increased image variations. During testing, bounding boxes were predicted by processing unknown ultrasound images through the trained DCNNs. A confidence score threshold of 0.20 was empirically set, with predicted bounding boxes below this threshold excluded from the results.

## 3. Experiments

For the validation of DCNN-based nerve detection, nerve detection experiments were conducted on the Public and Original datasets.

### 3.1. Experimental Setup

To evaluate the effects of model scaling and input image size scaling, we utilized DCNN models with different scales (i.e., YOLOv4-CSP, -P5, -P6, and -P7) and ultrasound images of varying sizes (i.e., 384 × 384, 640 × 640, 896 × 896, and 1152 × 1152 pixels). In each of the Public and Original datasets, a 20-fold cross validation was performed on images with each size for each DCNN model. In each fold, for the Public dataset, 600 ultrasound images, excluding 19 images (validation sub-dataset), were divided into 569–572 images (training sub-dataset) and 28–31 images (test sub-dataset) to encompass all types of nerves (i.e., the sciatic, ulnar, femoral, and median nerves) in the training, validation, and test sub-datasets. For the Original dataset, 939 images from 95 cases, excluding 54 images from 6 cases (validation sub-dataset), were divided into 871–911 images from 90–91 cases (training sub-dataset) and 28–68 images from 4–5 cases (test sub-dataset). The validation sub-datasets were empirically set to the minimum number of cases (3–5% of all cases) because we preliminarily confirmed that no significant overfitting occurred in the Public and Original datasets, where the imaging targets were somewhat limited. Although the test sub-dataset was reserved and the remaining data were allocated to the training and validation sub-datasets in a normal cross-validation, in this study, the validation sub-dataset was reserved, and the remaining data were allocated to training and test sub-datasets in each dataset to maximize the amount of training and test data available for evaluating the DCNN models.

In each dataset, the DCNN models were trained on the training sub-datasets with a batch size of 32 for up to 100 epochs. Following training, the best trained models were selected based on their performance on the validation sub-dataset and used to predict bounding boxes in the test sub-datasets. In the Original dataset, although the DCNN models were trained with seven classes, including nerves, blood vessels, and muscles, we evaluated the detection performance of the DCNNs focusing only on nerves and blood vessels, which are more critical detection targets.

The DCNN models were implemented using Python 3.8.0, OpenCV 4.6.0, and Pytorch 1.8.0 on Ubuntu 20.04.4 LTS. The experiments were conducted using NVIDIA CUDA 11.1.1 and cuDNN 8.0.5 on a workstation computer with dual AMD EPYC 7413 24-Core Processors, 1TB RAM, and eight NVIDIA A100 GPUs. The training of the DCNNs was performed on the eight GPUs, while the inference of the DCNNs was carried out on one of the GPUs.

### 3.2. Evaluation Metrics

**Figure 4:**
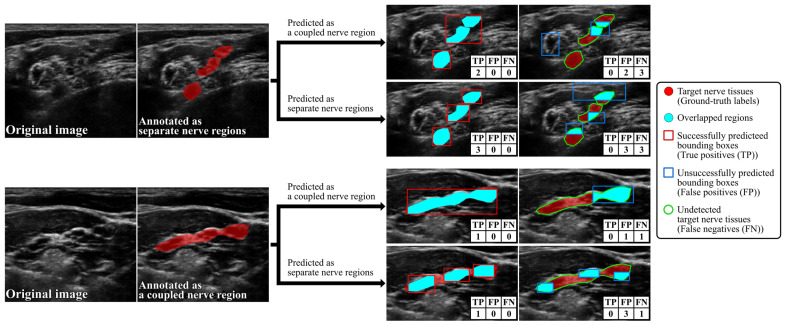
Examples of how to count true positives (TP), false positives (FP), and false negatives (FN) in the Original dataset.

The detection performance of the DCNN models was evaluated by analyzing overlaps between target tissues (ground-truth regions) and predicted bounding box regions. Initially, we employed the intersection over ground truth (IoGT) and intersection over bounding box (IoBB) metrics to categorize and count predicted bounding boxes as either successful or unsuccessful detections. The IoGT and IoBB are defined as follows:

(1) $$IoGT=\frac{\left|G\cap P\right|}{\left|G\right|},\;IoBB=\frac{\left|G\cap P\right|}{\left|P\right|}$$

where G and P denote pixels in the ground-truth region and the predicted bounding box region, respectively. The IoGT threshold was set at 0.5, indicating that a predicted bounding box contains the centroid of a ground-truth region. The IoBB threshold was set at 0.15, based on the minimal IoBB value observed between ground-truth regions and their corresponding bounding boxes, to identify if any bounding box predominantly encompasses the background. Consequently, predicted bounding boxes with IoGT≥0.5 and IoBB≥0.15 were counted as successful detections (i.e., true positives), while others were counted as unsuccessful detections (i.e., false positives). However, the Original dataset covers the brachial plexus, which has a network of nerves. In some instances, the nerve tissues are annotated separately, while in others, they are annotated as a coupled nerve region when in close proximity since it is difficult to establish a consistent criterion for annotating nerve tissues as a coupled nerve region or as separate nerve regions. The nerve tissues annotated as separate nerve regions by a physician may be predicted as a coupled nerve region by DCNNs and vice versa. In such cases, DCNN predictions should be considered correct. Therefore, as shown in Figure 4, when a predicted bounding box P overlapped multiple ground-truth regions $G_{i}\left(i=1,\;2,\ldots ,\;n\right)$ (i.e., when the DCNNs predicted separately annotated nerve tissues as a coupled nerve region), its detection success or failure was determined using the following ${IoGT}_{i}\left(i=1,\;2,\ldots ,\;n\right)$ and IoBB:

(2) $${IoGT}_{i}=\frac{\left|G_{i}\cap P\right|}{\left|G_{i}\right|},\;IoBB=\frac{\left|\left({⋃}_{i=1}^{n}G_{i}\right)\cap P\right|}{\left|P\right|}.$$

A predicted bounding box P with IoBB≥0.15 and ${IoGT}_{i}\geq 0.5$ for at least one ground-truth region $G_{i}$ was counted as a true positive, while ground-truth regions $G_{i}$ with ${IoGT}_{i}<0.5$ were counted as false negatives. Additionally, when multiple bounding boxes $P_{j}\left(j=1,\;2,\ldots ,\;m\right)$ overlapped the same ground-truth region G (i.e., when DCNNs predicted conjointly annotated nerve tissues as separate nerve regions), their detection success or failure was determined using the following IoGT and ${IoBB}_{j}\left(j=1,\;2,\ldots ,\;m\right)$:

(3) $$IoGT=\frac{\left|G\cap \left({⋃}_{j=1}^{m}P_{j}\right)\right|}{\left|G\right|},\;{IoBB}_{j}=\frac{\left|G\cap P_{j}\right|}{\left|P_{j}\right|}.$$

Even if each predicted bounding box $P_{j}\left(j=1,\;2,\ldots ,\;m\right)$ with $an\;{IoBB}_{j}\geq 0.15$ had an overlap of less than 50% of the ground-truth region G, the bounding boxes $P_{j}$ were counted as a true positive if IoGT≥0.5 (i.e., the total overlapped region for the bounding boxes was greater than or equal to 50% of the ground-truth region), and they were all counted as false positives if the IoGT<0.5. Finally, to quantify the detection accuracy based on the number of successful and unsuccessful detections, we used Recall, Precision, and the F1-measure, which were defined as follows:

(4) $$Recall[\%]=\frac{TP}{TP+FN}\times 100,$$

(5) $$Precision[\%]=\frac{TP}{TP+FP}\times 100,$$

(6) $$\text{F1-measure}=\frac{2\times Precision\times Recall}{Precision+Recall},$$

where TP, FP, and FN indicate true positives, false positives, and false negatives, respectively.

Furthermore, we measured the processing time from the input of ultrasound images to the inference and final output of bounding boxes, evaluating the feasibility of real-time DCNN-based nerve detection under US-PNB.

### 3.3. Results

**Figure 5:**
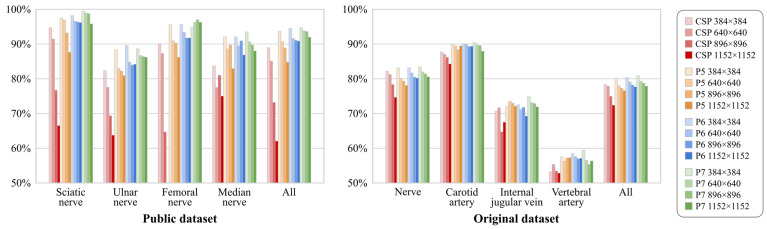
Detection results (F1-measure [%]) for anatomical structures identified with YOLOv4-CSP, -P5, -P6, and -P7 trained on input images of 384 × 384, 640 × 640, 896 × 896, and 1152 × 1152 pixels on the Public and Original datasets.

We compared anatomical structure detection results among DCNN models with different scales (i.e., YOLOv4-CSP, -P5, -P6, and -P7), trained on input images of varying sizes (i.e., 384 × 384, 640 × 640, 896 × 896, and 1152 × 1152 pixels), used on both the Public and Original datasets. Table 1 and Table 2 summarize the anatomical structure detection results (Recall and Precision) obtained on the Public and Original datasets, respectively. Figure 5 shows the anatomical detection results for the F1-measure as bar graphs. First, focusing on the detection results from DCNNs with different network scales, we found that networks with deeper scales achieved higher detection accuracies in both datasets; notably, the larger models, YOLOv4-P5, -P6, and -P7, tended to reduce over-detections and improve Precision compared to YOLOv4-CSP. Second, focusing on the detection results for different input image sizes, we noted that in both datasets, the use of smaller input images improved detection accuracy across all networks; specifically, it tended to decrease oversights and enhance Recall in nerve tissues. Consequently, YOLOv4-P7 trained on 384×384-pixel input images showed the highest F1-measure, achieving 94.7% for four types of nerve tissue in the Public dataset and 80.8% for anatomical structures including nerve and vascular tissues in the Original dataset.

**Figure 6:**
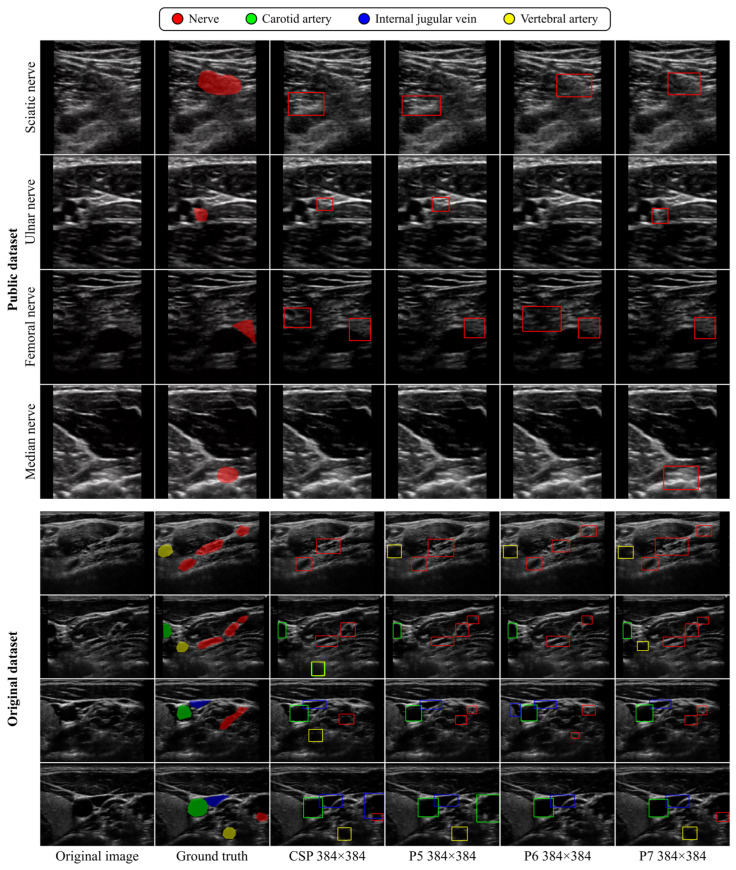
Visual comparison of detection results among YOLOv4-CSP, -P5, -P6, and -P7 trained on 384 × 384-pixel input images in the Public and Original datasets.

**Figure 7:**
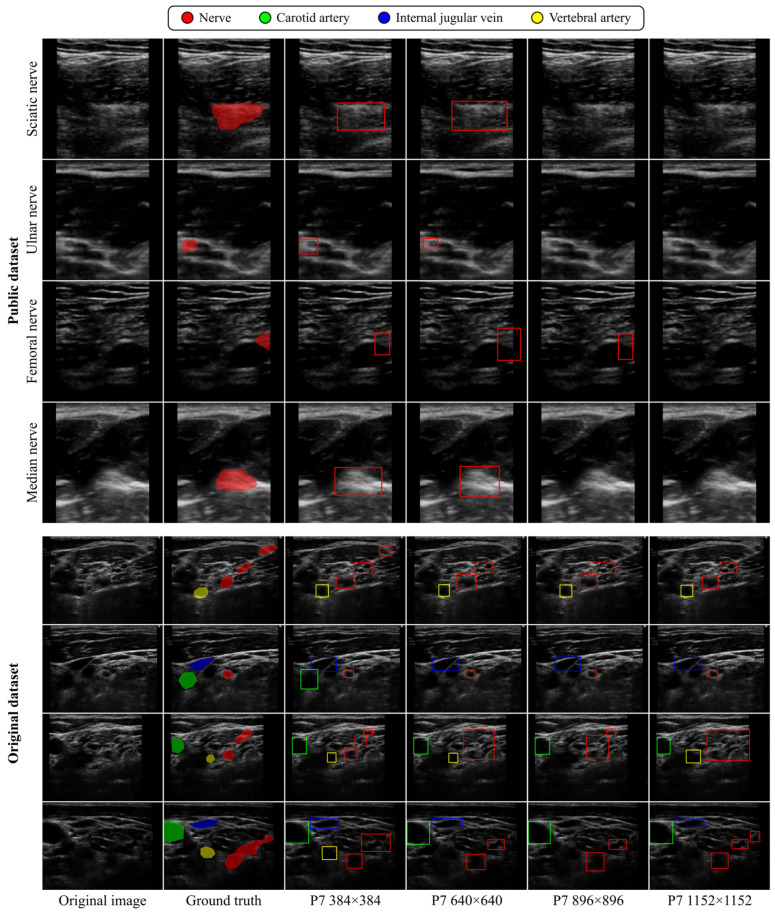
Visual comparison of detection results among YOLO-P7 models trained on input images of 384 × 384, 640 × 640, 896 × 896, and 1152 × 1152 pixels in Public and Original datasets.

For visual comparisons, Figure 6 illustrates the detection results from DCNN models of different scales trained on 384 × 384-pixel input images, while Figure 7 visualizes the detection results from the YOLOv4-P7 model trained on input images of varying sizes. These figures present images with relatively low positive detection rates in sixteen patterns of detection results consisting of combinations of four different model sizes and four different image sizes. As depicted in Figure 6, we noted that the larger DCNN models improved the over-detection of structures confusable with tubular structures and the oversight of anatomical structures, which occurred when the smaller DCNN models were used. Additionally, as illustrated in Figure 7, we observed instances in which the use of smaller input images enabled the DCNNs to capture anatomical structures that were overlooked when larger input images were used.

Table 3 indicates the inference times for DCNN models of varying scales and input image sizes in the Original dataset. As expected, the inference time correlated positively with both the scale of the DCNN models and the size of the input images. Specifically, the inference time for YOLOv4-P7, which had the most parameters, was approximately 2–3 times longer than that for YOLOv4-CSP, which had the fewest parameters. Similarly, the inference time for the largest input images, which measured 1152 × 1152 pixels, was about 5–8 times longer than for the smallest input images, measuring 384 × 384 pixels. The combination of YOLOv4-P7 and 384 × 384-pixel input images, which achieved the highest detection accuracy, resulted in an inference time of 5.2 milliseconds (ms), which is equivalent to 192.4 frames per second (fps), inclusive of the time for maximum suppression post processing.

## 4. Discussion

We verified the feasibility of DCNN-based nerve detection for US-PNB, evaluating the effects of DCNN model size and input image size on the detection of nerves and their surrounding blood vessels. Regarding model size, the largest DCNN model (i.e., YOLOv4-P7) demonstrated superior detection performance, as reported in a previous study on general object detection tasks [25]. Scaling up the DCNN model size resulted in fewer over-detections and a marked improvement in Precision. This enhancement is likely attributable to the larger models’ capacity to discern more complex and abstract image features across multiple scales. Although over-detection often occurs on ultrasound images due to the appearance of tube-like structures resembling nerve and vascular tissues, a larger model with advanced feature extraction capabilities is expected to mitigate this issue. Conversely, concerning input image size, the smallest input images (i.e., 384 × 384-pixel input images) yielded the best detection performance, despite a previous study [25] suggesting that larger input images with larger models enhance detection accuracy. This discrepancy may stem from the unique characteristics of ultrasound images, which often contain speckle noise that becomes more pronounced at higher resolutions, thereby hindering accurate structure detection. By reducing the resolution, the fine noise is smoothed, diminishing its impact and consequently enhancing detection performance. Experimental results indicate that reducing resolution improves Recall, suggesting that fine noise in ultrasound images may lead to overlooked nerve and vascular tissues and that image downscaling can help reduce such oversights.

The combination of YOLOv4-P7 and 384 × 384-pixel input images exhibited the highest nerve-detection performance among the tested model and image size configurations, achieving detection accuracies exceeding 90% for the Public dataset and 80% for the Original dataset. As detailed in Table 3, this combination also had an inference time of 5.2 ms (192.4 fps). Although this time does not account for pre-processing tasks such as image loading and resizing, detection speeds of 30–60 fps, inclusive of pre-processing, are generally sufficient for real-time display during US-PNB. Thus, the real-time detection of anatomical structures appears feasible with this optimal model and image size configuration, supporting the use of DCNN-based nerve detection to aid anesthesiologists during US-PNB.

However, this study has some limitations. First, despite utilizing ultrasound images from two datasets, the sample size was still small and may not adequately cover the variability inherent in ultrasound images influenced by factors such as patient variability, probe manipulations, and device settings. Thus, we will need to use more ultrasound images acquired under various conditions to enhance the generalization performance of DCNNs and ensure more rigorous validation. Specifically, as the ultrasound images in each of the Public and Original datasets were collected using a single ultrasound device at one medical facility, we will need to examine the effect of different devices or different probe techniques based on images acquired with multiple ultrasound devices by more anesthesiologists across different facilities. Second, this study focused solely on detecting anatomical structures from ultrasound images. Since anesthesiologists need to identify not only anatomical structures but also the needle on ultrasound images during procedures, it would be desirable to automatically detect both the needle and anatomical structures to enhance the safety of US-PNB. Third, while this study successfully elucidated the performance of a one-stage DCNN in detecting nerve tissues from ultrasound images along with optimal combinations of model scaling and input image scaling, it does not provide a comparative evaluation of different DCNN architectures. As indicated in previous studies [2,24], integrating custom modules designed for the specific task of detecting nerves from ultrasound images can significantly enhance detection performance. Similarly, YOLO-based DCNNs continue to evolve, incorporating various module improvements to boost both accuracy and speed [34]. Hence, although our findings suggest that a larger DCNN model with smaller input images improves the performance of nerve detection in ultrasound images, which is an important insight likely applicable to other DCNN models, it will be crucial to compare the performance of different DCNN model structures to investigate effective modules for nerve detection in ultrasound images. Additionally, this study focused on input image scaling, demonstrating that reducing input image scale can potentially mitigate the impact of speckle noise and improve detection performance. However, denoising techniques, such as despeckle filtering [5] or deep learning-based denoising [19], are also reported in the literature as effective approaches. Therefore, it is important to investigate whether input image scaling or denoising is more effective for performance enhancement or if a combination of both methods could lead to further improvements in detection accuracy.

## 5. Conclusions

This paper presents DCNN-based nerve detection in ultrasound images, aiming to assist anesthesiologists in localizing nerve tissues during US-PNB. Utilizing Scaled-YOLOv4, a scalable DCNN model for object detection, we explored various configurations of DCNN model sizes and input image sizes. Our findings indicate that larger models paired with smaller input images offer the best balance between accuracy and speed, achieving high detection performance with real-time processing capabilities. The experimental results demonstrated that the optimal configuration, YOLOv4-P7 with 384 × 384-pixel input images, could detect nerve tissues with a high F1-measure of over 80% and an inference speed of 192.4 fps, underscoring the feasibility of real-time DCNN-based nerve detection for US-PNB. Future work will focus on expanding the datasets to encompass greater variability, investigating more effective DCNN modules or denoising methods through comparative studies, and implementing the concurrent detection of both anatomical structures and procedural instruments, such as needles, to further enhance the safety and efficacy of US-PNB procedures.

## Figures and Tables

**Table 1:** Detection results (Recall [%] and Precision [%]) for nerve tissues with YOLOv4-CSP, -P5, -P6, and -P7 trained on input images of 384 × 384, 640 × 640, 896 × 896, and 1152×1152 pixels on the Public dataset. The best result for each class is shown in bold.

Network	Image Size [Pixels]	Class									
		Sciatic Nerve		Ulnar Nerve		Femoral Nerve		Median Nerve		All	
		Recall	Precision	Recall	Precision	Recall	Precision	Recall	Precision	Recall	Precision
CSP	384 × 384	96.4	93.1	86.0	79.0	92.6	87.5	**90.0**	78.3	91.8	86.2
	640 × 640	88.5	94.6	74.3	81.1	80.9	94.8	77.5	77.5	81.8	88.6
	896 × 896	66.2	91.1	59.8	82.6	48.5	97.1	80.0	82.1	62.8	87.7
	1152 × 1152	53.6	87.6	51.4	84.0	10.3	63.6	67.5	84.4	48.8	85.2
P5	384 × 384	97.5	97.5	87.4	89.5	**95.6**	95.6	87.5	97.2	93.0	94.4
	640 × 640	96.0	97.8	81.8	84.1	88.2	93.8	87.5	89.7	89.5	92.0
	896 × 896	91.4	95.1	75.7	90.0	88.2	92.3	87.5	92.1	85.2	92.9
	1152 × 1152	82.7	93.1	74.3	88.8	77.9	96.4	85.0	81.0	79.3	91.0
P6	384 × 384	97.8	98.6	**88.3**	**90.9**	**95.6**	95.6	87.5	97.2	93.5	95.4
	640 × 640	95.7	97.4	80.8	89.2	92.6	94.0	85.0	94.4	89.3	94.0
	896 × 896	95.0	97.8	81.8	86.2	89.7	93.8	87.5	94.6	89.2	93.0
	1152 × 1152	94.2	98.1	79.4	89.5	89.7	93.8	82.5	91.7	87.7	94.3
P7	384 × 384	**99.3**	**99.6**	87.4	89.9	94.1	95.5	**90.0**	**97.3**	**93.8**	95.6
	640 × 640	98.9	98.9	83.6	89.9	94.1	98.5	85.0	97.1	92.0	**95.7**
	896 × 896	98.2	99.3	84.6	88.3	94.1	**100.0**	87.5	92.1	92.2	95.0
	1152 × 1152	93.9	97.8	83.2	89.4	94.1	98.5	82.5	94.3	89.3	94.7

**Table 2:** Detection results (Recall [%] and Precision [%]) for nerve and vascular tissues with YOLOv4-CSP, -P5, -P6, and -P7 trained on input images of 384 × 384, 640 × 640, 896 × 896, and 1152 × 1152 pixels on the Original dataset. The best result for each class is shown in bold.

Network	Image Size [Pixels]	Class									
		Nerve		Carotid Artery		Internal Jugular Vein		Vertebral Artery		All	
		Recall	Precision	Recall	Precision	Recall	Precision	Recall	Precision	Recall	Precision
CSP	384 × 384	**82.9**	81.6	94.6	81.7	78.9	64.0	60.9	47.3	**81.5**	75.5
	640 × 640	81.9	80.6	93.4	81.5	**81.3**	64.1	**64.8**	48.3	81.2	74.8
	896 × 896	76.8	80.0	94.3	79.3	77.8	55.4	63.5	46.1	77.4	72.7
	1152 × 1152	74.3	75.1	93.7	76.5	78.4	59.3	**64.8**	44.6	75.8	69.3
P5	384 × 384	80.4	**86.1**	93.4	86.5	76.6	68.2	57.3	57.7	79.0	81.4
	640 × 640	78.0	82.5	92.7	86.5	73.7	**73.3**	57.7	55.0	77.1	78.9
	896 × 896	75.1	84.1	93.1	84.0	76.6	69.7	60.6	54.2	75.7	78.9
	1152 × 1152	73.8	82.8	92.4	86.7	72.5	71.7	58.3	56.3	74.2	79.1
P6	384 × 384	80.8	85.7	91.8	**88.4**	80.1	66.2	58.3	**58.7**	79.4	81.2
	640 × 640	79.5	84.0	93.1	87.0	73.7	68.9	59.3	56.0	78.4	79.8
	896 × 896	77.6	83.6	93.4	85.5	76.6	67.5	57.0	57.0	77.0	79.4
	1152 × 1152	76.6	84.1	94.0	85.1	77.8	62.4	59.6	54.8	76.8	78.6
P7	384 × 384	82.4	84.6	**95.3**	86.0	80.7	69.7	61.2	57.8	81.3	80.4
	640 × 640	80.1	83.9	91.2	88.4	77.2	69.5	56.7	56.5	78.5	80.1
	896 × 896	77.3	86.0	91.8	87.4	77.8	68.6	53.1	57.8	76.2	**81.5**
	1152 × 1152	77.4	84.0	90.5	85.4	80.1	65.2	58.0	54.8	76.9	78.9

**Table 3:** Lengths of inference time of YOLOv4-CSP, -P5, -P6, and -P7 with input images of 384 × 384, 640 × 640, 896 × 896, and 1152 × 1152 pixels in Original dataset. Total time includes inference time and non-maximum suppression post processing time. ms: millisecond and fps: frame per second.

Network	Parameters	Image Size [Pixels]	Inference Time	Total Time
CSP	52.5 M	384 × 384	1.5 ms (687.2 fps)	2.0 ms (497.3 fps)
		640 × 640	3.8 ms (265.2 fps)	4.3 ms (231.2 fps)
		896 × 896	6.6 ms (152.6 fps)	7.1 ms (140.6 fps)
		1152 × 1152	12.0 ms (83.5 fps)	12.6 ms (79.7 fps)
P5	70.3 M	384 × 384	2.0 ms (507.7 fps)	2.5 ms (395.3 fps)
		640 × 640	4.8 ms (207.5 fps)	5.4 ms (185.1 fps)
		896 × 896	9.3 ms (107.9 fps)	9.8 ms (101.7 fps)
		1152 × 1152	14.3 ms (70.2 fps)	14.8 ms (67.4 fps)
P6	126.7 M	384 × 384	2.6 ms (385.2 fps)	3.2 ms (316.6 fps)
		640 × 640	5.7 ms (175.8 fps)	6.3 ms (159.9 fps)
		896 × 896	9.7 ms (103.5 fps)	10.2 ms (97.7 fps)
		1152 × 1152	15.3 ms (65.2 fps)	15.9 ms (62.7 fps)
P7	286.1 M	384 × 384	4.6 ms (216.4 fps)	5.2 ms (192.4 fps)
		640 × 640	10.5 ms (95.1 fps)	11.1 ms (90.1 fps)
		896 × 896	16.4 ms (61.1 fps)	17.0 ms (59.0 fps)
		1152 × 1152	25.6 ms (39.1 fps)	26.2 ms (38.2 fps)

## Data Availability

The data presented in this study are available upon request from the corresponding author. The data are not publicly available because of ethical restrictions.
